# Malignant Small Bowel Obstruction Unveiling Rare High-Grade Serous Ovarian Carcinoma of Mullerian Origin: A Case Report Detailing Diagnosis and Management

**DOI:** 10.7759/cureus.108592

**Published:** 2026-05-10

**Authors:** Riddhi Machchhar, Fatima Mossolem, Ahmed D Al Mahrizi, Sherein Bain, Alexandra Greenberg

**Affiliations:** 1 Internal Medicine, Ocean University Medical Center, Brick, USA; 2 Graduate Medical Education, Praxis Institute, Voorhees, USA; 3 Education, Futures Forward Research Institute, Toms River, USA; 4 Faculty of Medicine and Surgery, University of Malta, Msida, MLT; 5 Internal Medicine, St. Joseph’s Hospital-South, Riverview, USA; 6 Gastroenterology, Ocean University Medical Center, Brick, USA

**Keywords:** endoscopy, gastrojejunostomy, high-grade serous ovarian cancer, malignant small bowel obstruction, mullerian origin, push enteroscopy

## Abstract

Malignant small bowel obstruction in advanced ovarian carcinoma is uncommon but carries high morbidity; prompt diagnosis and targeted intervention are critical to relieve symptoms and maintain nutrition. This case is distinguished by an isolated obstruction at the third portion of the duodenum without a discrete intraluminal mass, an atypical site and mechanism in recurrent high-grade serous ovarian carcinoma, necessitating a tailored surgical approach. A 61-year-old female with an established diagnosis of high-grade serous ovarian carcinoma presented with acute-onset abdominal distension, nausea, and obstipation. Contrast-enhanced CT with oral contrast was not feasible due to the patient’s inability to tolerate oral intake; therefore, non-contrast imaging was obtained, demonstrating marked dilation of the stomach and proximal duodenum with a transition point at the third portion of the duodenum. Endoscopic decompression yielded 1,700 mL of bilious material; no discrete intraluminal lesion was identified, raising suspicion for extrinsic compression. Exploratory laparotomy confirmed peritoneal and mesenteric implants, with biopsy consistent with recurrent high-grade serous carcinoma. Given the absence of a focal intraluminal lesion amenable to stenting and the presence of multifocal extrinsic disease, a surgical bypass was favored over endoscopic palliation to provide more durable symptom relief and maintain enteral nutrition. Operations performed included gastrojejunal bypass and jejunostomy tube placement. Postoperatively, the patient tolerated nasogastric decompression and total parenteral nutrition, successfully transitioning to jejunal feeding. She ultimately resumed oral intake and was discharged home on postoperative day 17, with plans for adjuvant chemotherapy. This case highlights the importance of an interdisciplinary approach, incorporating early imaging, endoscopic evaluation, and individualized surgical decision-making, in managing malignant small bowel obstruction in ovarian cancer. Recognition of atypical obstruction sites and mechanisms can guide appropriate intervention, improving gastrointestinal function and overall quality of life.

## Introduction

Malignant bowel obstruction (MBO) is an uncommon pathology in which a neoplasm causes mechanical obstruction of the gastrointestinal (GI) tract, often associated with high morbidity and mortality. In the United States, the overall mortality rate of this unique pathology was 21.4% in 2010, with the incidence only expected to rise [1]. As per the National Institute of Health (NIH)’s National Cancer Institute’s Surveillance, Epidemiology, and End Results data, the most common malignant causes of MBOs are GI and ovarian cancers, respectively. GI malignancies have a higher prevalence, and ovarian pathology is the leading cause of death among other gynecological malignancies [2]. The global incidence of MBO is 3-15% of cancer patients, but can reach 20-50% in patients with malignancy of ovarian origin [3]. An international consensus group has proposed the following standardized diagnostic criteria for MBO: (a) clinical evidence of bowel obstruction, (b) obstruction distal to the ligament of Treitz, (c) the presence of primary intra-abdominal or extra-abdominal malignancy with peritoneal involvement, and (d) absence of reasonable curative options [3]. Despite its recognized association with advanced ovarian cancer, duodenal involvement is rare and poses unique diagnostic and therapeutic challenges. This report aims to highlight an atypical presentation of ovarian cancer-related MBO at this location, emphasizing the importance of precise diagnostic evaluation and individualized, multidisciplinary management.

## Case presentation

**Figure 1:**
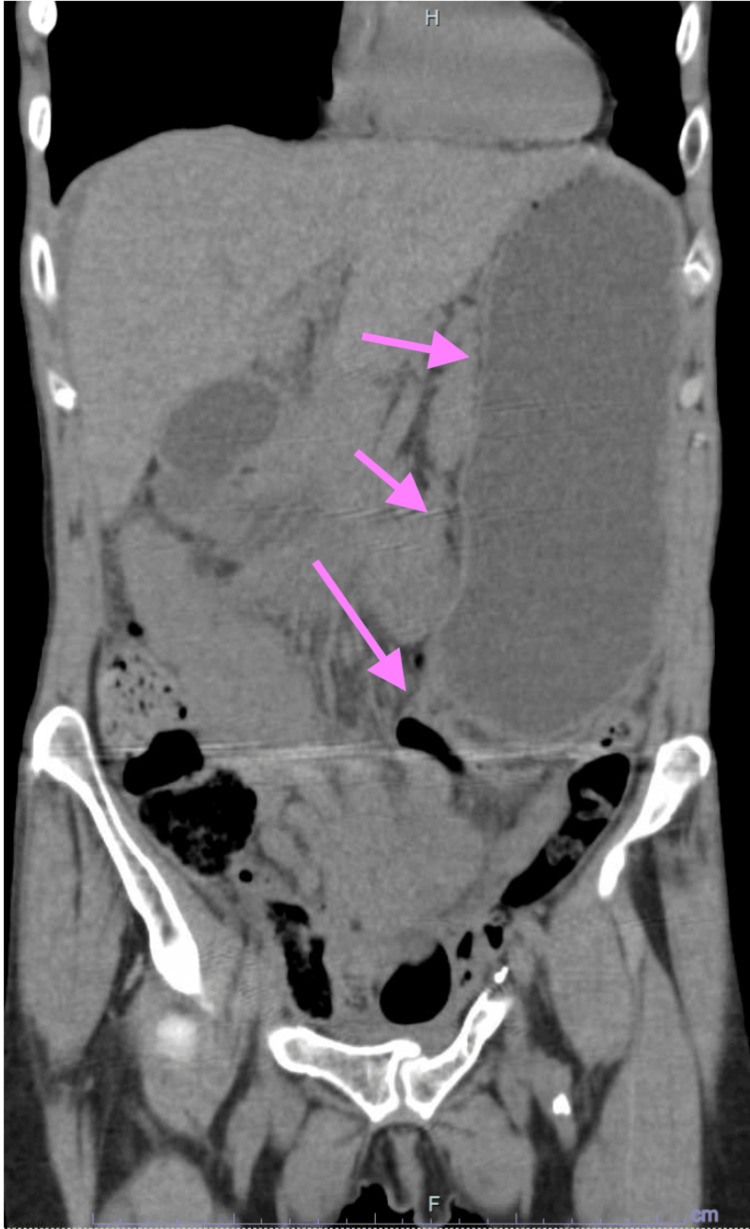
CT of the abdomen/pelvis without contrast showing significant distention of the stomach and proximal-mid duodenum. There is a changing caliber as the duodenum crosses the inferior vena cava. Evaluation for masses limited without intravenous contrast.

A 61-year-old female presented to the emergency department (ED) with bloating, nausea, and vomiting. Her symptoms first began one and a half weeks prior with a new-onset epigastric and substernal burning associated with dyspepsia, which spontaneously resolved, but later returned after ingestion of spicy food. Past medical history was significant for tobacco use disorder and removal of an ovarian teratoma. The patient attempted to treat the epigastric burning with over-the-counter esomeprazole, which yielded marginal relief for one day. However, the patient reported feeling a rapidly progressive acidity and a lump in her throat. These symptoms were exacerbated on lying supine and eating. The patient also reported fatigue, nausea, and six to seven episodes of bilious emesis refractory to calcium carbonate and sodium bicarbonate. The vomitus progressed to a dark brown emesis and was accompanied by significant abdominal distension. Before presentation, the patient limited her diet to Pedialyte and Ensure but still noted worsening abdominal distention. A CT scan of the abdomen and pelvis without contrast was performed in the ED, which showed significant distension of the stomach and the proximal mid-duodenum. There was a changing caliber as the duodenum crossed the inferior vena cava with moderate constipation (Figure 1).

**Figure 2:**
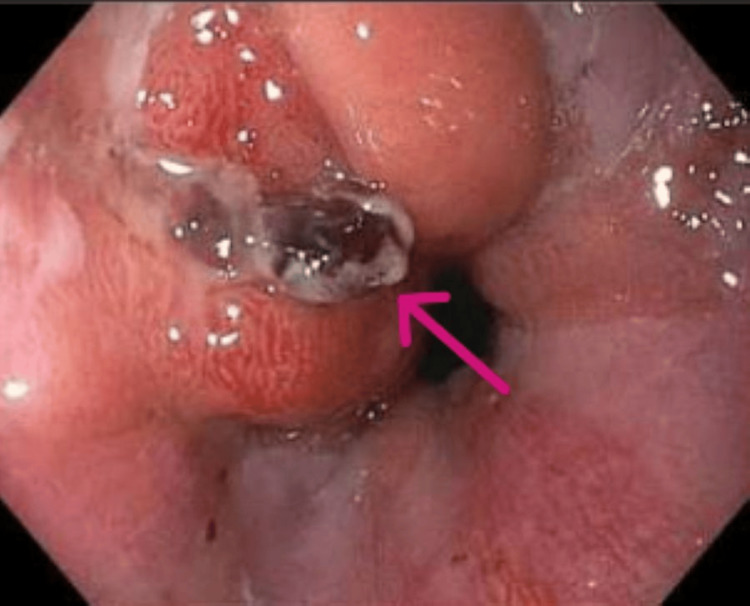
First endoscopy depicting a Mallory-Weiss tear.

Initial placement of a nasogastric tube (NGT) yielded 1 L of black fluid; however, once successfully placed, the patient’s vomiting was again triggered. Further history was collected, and the patient denied use of nonsteroidal inflammatory drugs, dyspepsia with food intake, weight loss, loss of appetite, hematochezia, melena, and constipation. The patient was subsequently scheduled for an endoscopy (esophagoduodenoscopy, EGD), which revealed a healing Mallory-Weiss tear (Figure 2), a small hiatal hernia, but no retained food, and no gastric outlet obstruction.

**Figure 3:**
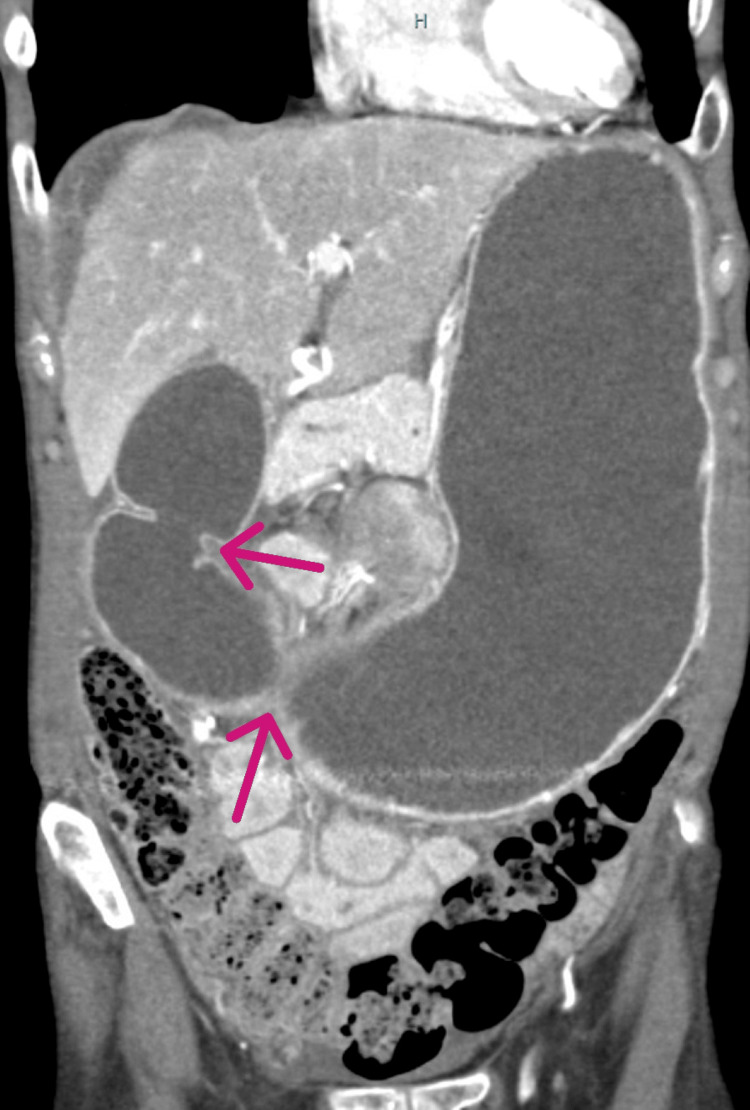
CT enterography showing distention of the stomach with oral contrast. There is marked distention of the duodenal bulb and the second portion of the duodenum. The stomach is found below the third and fourth portions of the duodenum, which do not appear to cross the midline, suggesting malrotation. No oral contrast has extended beyond the second portion of the duodenum. There is a collapse of the remainder of the small bowel. Scattered mild stool is seen throughout the colon. While a mass is not identified, a mass in the third portion of the duodenum cannot be excluded. Direct visualization was recommended by the radiologist.

The patient was only able to tolerate two days of a liquid-to-soft diet before her symptoms of nausea, bilious vomiting, and distention returned. A stat CT enterography was performed, which showed a massively distended stomach and a questionable mass in the third portion of the duodenum. The study provided limited insight, and malrotation was suspected (Figure 3).

Thus, she underwent a second EGD after a failed reinsertion of an NGT. During this procedure, 1,700 cc of bilious fluid was removed from the stomach; no discrete mass was appreciated, but biopsies were performed to rule out Barrett’s esophagus. An orogastric tube (OGT) was placed at this time with settings set to low-suction.

**Figure 4:**
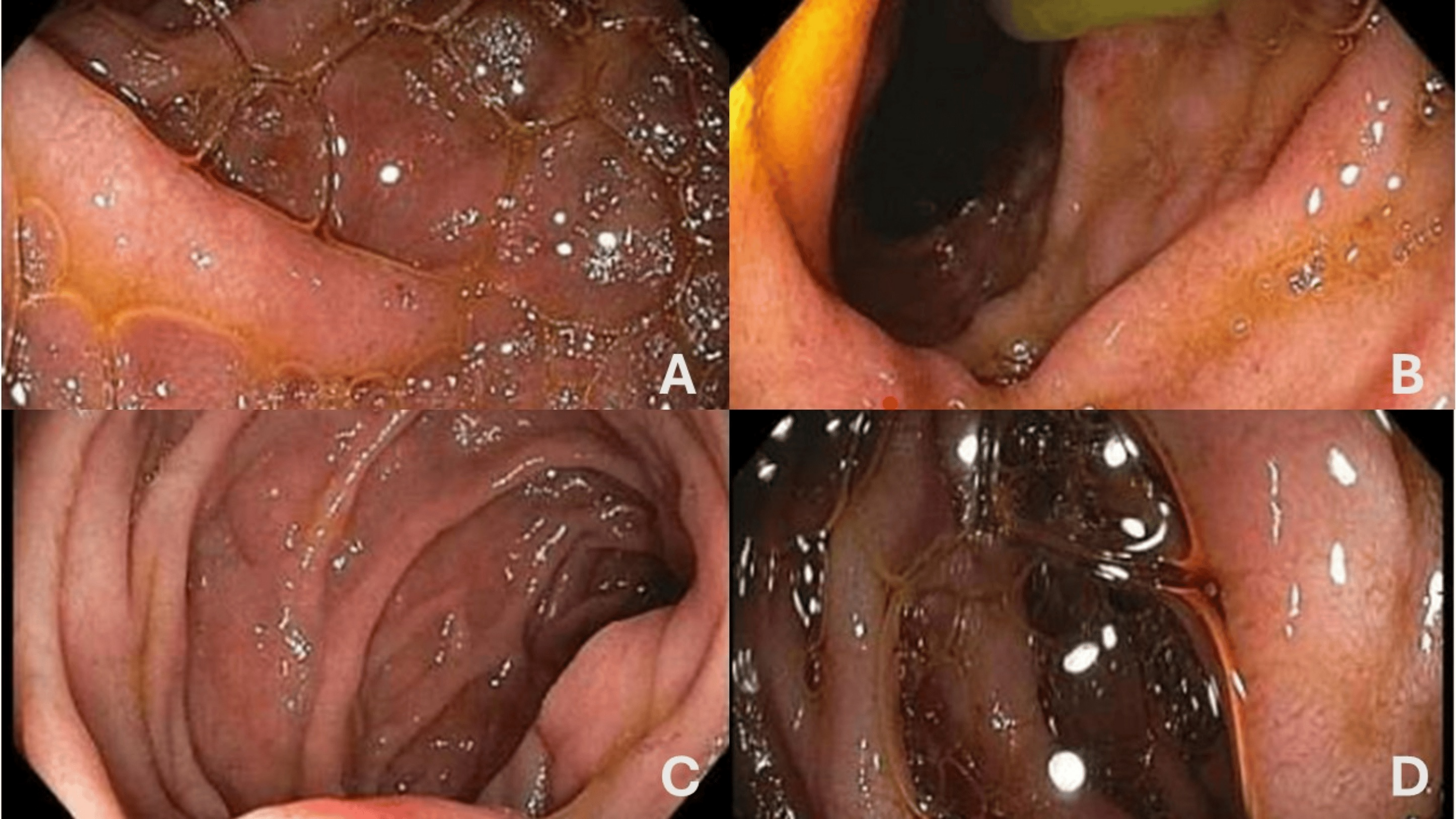
Enteroscopy showing the (a) duodenal bulb, (b) second part of the duodenum, and (c, d) fourth portion of the duodenum. A deformity can be appreciated in the third part of the duodenum, suspicious of narrowing/stenosis and/or mass.

General surgery was consulted to coordinate further care with the gastroenterologist, and a push enteroscopy was recommended and performed. These results were similar to both EGDs and noted a stenosis at the third part of the duodenum (Figure 4).

**Figure 5:**
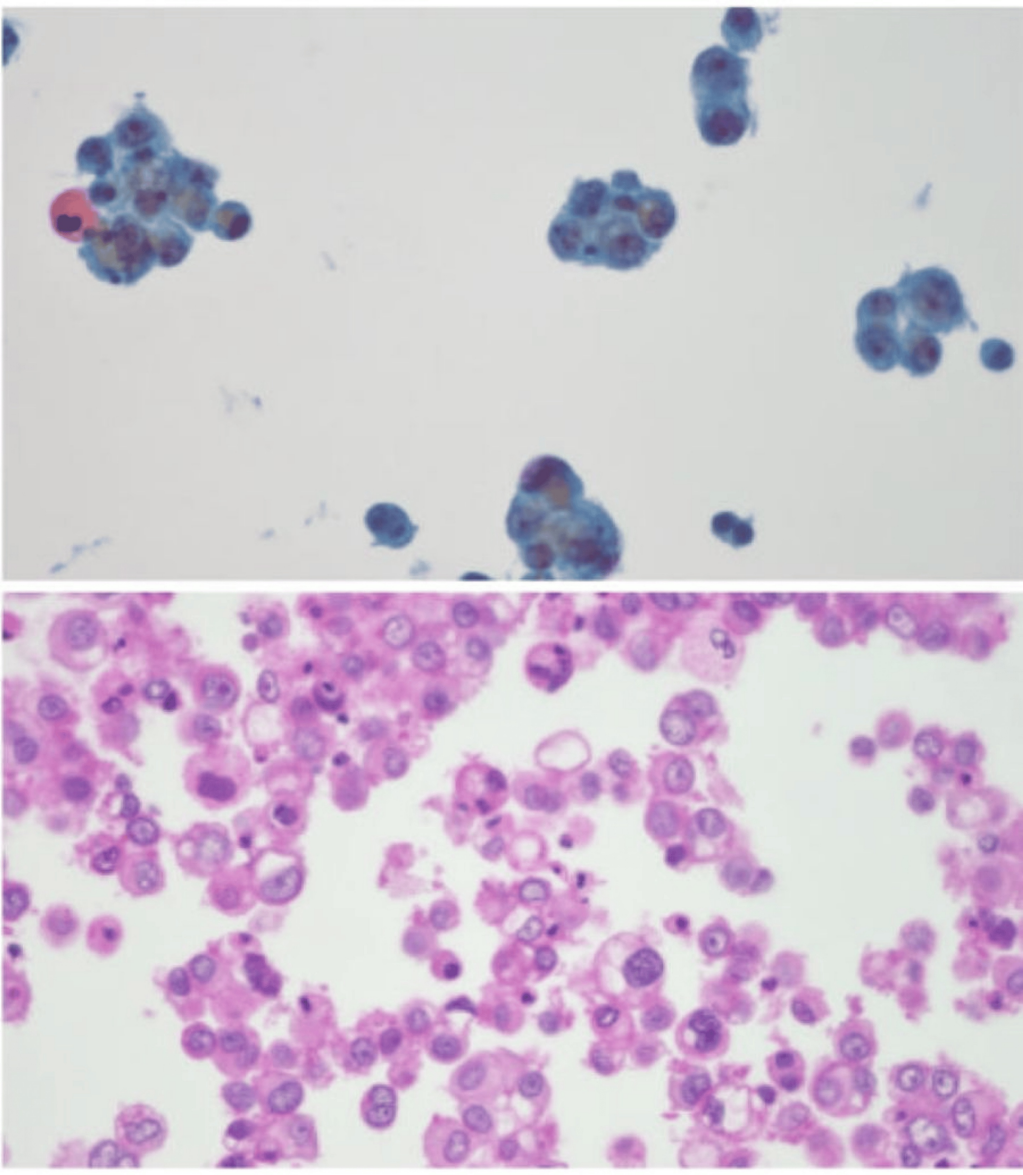
Immunohistochemistry slide displaying high-grade carcinoma, favoring serous carcinoma of Mullerian origin.

During this time, the patient received a peripherally inserted central catheter line for initiation of total parenteral nutrition (TPN) and provided supplemental management. The following day, the patient underwent an ex-lap with excisional biopsy of the peritoneal implant, mesenteric implant, and enlarged perigastric lymph node. Additionally, a gastrojejunal bypass with a feeding jejunostomy (J-tube) was performed. Post-operation, the patient remained with an OGT set to low continuous suction while TPN was maintained until enteral trickle feeds could be initiated via the J-tube. Hematology-Oncology was consulted in light of the ex-lap findings. The patient’s ex-lap results returned with carcinomatosis consisting of high-grade carcinoma, favoring serous carcinoma of Mullerian origin (Figure 5).

The remainder of the patient’s admission focused on clamp trials in light of persistent bilious OGT output, transitioning to tube feeds at goal, and eventually insertion of a percutaneous gastrostomy tube for gastric decompression. She was successfully discharged with a plan for chemotherapy, tube feeds at goal, and home care (Table 1).

**Table 1:** Timeline of clinical events and interventions.

Date	Investigation/Procedure	Key findings/Interventions
Day 0	CT of the abdomen and Pelvis (no contrast)	Marked distension of the stomach and proximal duodenum; caliber change at the inferior vena cava crossing (Figure 1)
Day 0	Nasogastric tube placement	1 L black fluid drained; symptoms briefly improved
Day 2	Esophagoduodenoscopy (EGD #1)	A Mallory–Weiss tear at the gastroesophageal junction; no obstruction; small hiatal hernia (Figure 2)
Day 5	CT enterography	Massive gastric distension; possible third-portion duodenal mass or malrotation (Figure 3)
Day 5	Esophagoduodenoscopy (EGD #2)	1,700 cc bilious fluid removed; biopsies taken; no discrete mass
Day 8	Push enteroscopy	Stenosis/Deformity in the third part of the duodenum; mass suspected (Figure 4)
Day 9	Exploratory laparotomy with excisional biopsies and bypass	Peritoneal/Mesenteric implants and lymph node biopsied; high-grade serous carcinoma diagnosed; gastrojejunal (GJ) bypass and GJ-tube placed (Figure 5)
Days 10–16	Postoperative supportive care	Low-suction oral gastric tube, total parenteral nutrition → transition to jejunal feeds via J-tube
Day 17	Percutaneous endoscopic gastrostomy (PEG) placement	Decompression established; preparation for discharge
Day 19	Discharge	Home care with PEG decompression, jejunal feeds, oncology follow-up and chemotherapy plan

## Discussion

The most common causes of small bowel obstruction (SBO) are adhesions (70-75%) and malignancy, such as this case (5% to 20%) [4-6]. The diagnosis of SBO in our patient relied on a combination of past medical history and physical examination findings. In this case, diagnosis was guided by the patient’s oncologic history, clinical presentation, and imaging findings. Initial evaluation with abdominal radiography and CT demonstrated proximal bowel dilation with a transition point, raising concern for obstruction. While ovarian cancer can present with nonspecific GI symptoms such as bloating, ascites, and abdominal pain, it may also manifest acutely as malignant SBO (mSBO), particularly in advanced disease [7]. Importantly, the first presentations of mSBO often lead to surgical intervention [8].

mSBO often arises from peritoneal disease and carcinomatosis, typically due to metastatic disease from the abdomen or pelvis, typically leading to adhesions or obstructions from the ligament of Treitz to the ileocecal valve [8]. Primary intra-abdominal malignancies can spread through local advancement, causing intraluminal obstruction or extraluminal compression [8]. They may also disrupt the innervating plexi, leading to paresis and subsequently mSBO [7,8]. Duodenal involvement is uncommon and represents a distinct diagnostic and therapeutic challenge, as seen in this case. Other causes of SBO include prior abdominal surgery (70%), hernias (10%), and irritable bowel disease (5%) [9].

In a 10-year retrospective cohort study, the mean age of mSBO patients was 67 years, predominantly affecting females [8]. Most cases of mSBO presented emergently, with 89% dying within 10 years and 15% within one month [7,8]. Many admissions had peritoneal metastatic disease alongside ovarian, retroperitoneal, and bowel metastases, with others experiencing primary cancer-causing SBO [8]. Most of these primary cancers had mechanical obstructions due to locally advanced diseases, while some had advanced disease within the small bowel [7,8]. Prognosis remains poor, particularly in the setting of peritoneal metastatic disease, underscoring the importance of individualized, goal-directed management. However, reported outcomes vary widely across studies, and survival is influenced by factors such as disease burden, functional status, and treatment approach rather than obstruction etiology alone [7,8].

Management of mSBO requires a stepwise and multidisciplinary approach. Initial treatment is typically conservative, including bowel rest, nasogastric decompression, and fluid resuscitation. In this patient, the transition from conservative to surgical management was driven by persistent obstruction, inability to tolerate oral intake, and imaging and endoscopic findings suggestive of a fixed transition point without a targetable intraluminal lesion. While endoscopic stenting may be considered in select cases, it is often limited in proximal or extrinsically compressed lesions. Given the patient’s duodenal obstruction from multifocal peritoneal disease, surgical gastrojejunal bypass was selected to provide more durable symptomatic relief and enable enteral nutrition. The role of adjunctive therapies in mSBO is nuanced. TPN may serve as a temporary supportive measure in carefully selected patients, but does not independently improve survival and should be aligned with the overall goals of care. Similarly, systemic chemotherapy may be considered in patients with chemosensitive disease, but it is not an acute intervention for obstruction and should be individualized. Corticosteroids may provide short-term symptomatic relief but have a limited impact on long-term outcomes [8].

High-grade serous ovarian carcinoma, the most common subtype of ovarian cancer, is characterized by aggressive behavior and a propensity for peritoneal dissemination, predisposing patients to mSBO [10]. This case highlights several key learning points: the atypical location of obstruction at the third portion of the duodenum, the diagnostic challenge posed by the absence of an intraluminal lesion, and the importance of integrating imaging, endoscopic evaluation, and surgical judgment in management. Compared with existing literature, which predominantly describes distal small bowel involvement, this case underscores the need to consider proximal obstruction and tailor interventions accordingly.

Overall, this case reinforces the importance of a multidisciplinary, patient-centered approach in mSBO, particularly in rare presentations such as duodenal obstruction in ovarian cancer, where individualized decision-making is essential to optimize symptom control and quality of life.

## Conclusions

In patients with SBO, it is imperative for clinicians to identify the underlying cause, particularly when malignancy is suspected. The acute onset of generalized GI symptoms such as abdominal pain, nausea, and vomiting in patients without prior similar symptoms should prompt evaluation for an underlying pathology. This case highlights malignant duodenal obstruction as an uncommon presentation of recurrent high-grade serous ovarian carcinoma, emphasizing the diagnostic challenge of a proximal obstruction without a discrete intraluminal lesion. When treating malignancy, it is also important to ensure patient comfort and adherence to the overall treatment plan. Our case underscores the value of early multidisciplinary management, including radiologic, endoscopic, and surgical approaches, in guiding timely intervention and optimizing symptom control. This case serves as a reminder that bowel obstruction can have a variety of causes and that patients presenting with nonspecific symptoms should be closely monitored and followed until resolution, particularly when atypical obstruction sites are involved.
